# Controlling the familywise error rate in widefield optical neuroimaging of functional connectivity in mice

**DOI:** 10.1117/1.NPh.10.1.015004

**Published:** 2023-02-03

**Authors:** Brian R. White, Claudia Chan, Temilola Adepoju, Russell T. Shinohara, Simon Vandekar

**Affiliations:** a University of Pennsylvania, Children’s Hospital of Philadelphia, Perelman School of Medicine, Division of Cardiology, Department of Pediatrics, Philadelphia, Pennsylvania, United States; b University of Pennsylvania, Perelman School of Medicine, Department of Biostatistics, Epidemiology, and Informatics, Philadelphia, Pennsylvania, United States; c University of Pennsylvania, Center for Biomedical Image Computing and Analysis, Department of Radiology, Philadelphia, Pennsylvania, United States; d University of Pennsylvania, Penn Statistics in Imaging and Visualization Endeavor, Department of Biostatistics, Epidemiology, and Informatics, Philadelphia, Pennsylvania, United States; e Vanderbilt University, Department of Biostatistics, Nashville, Tennessee, United States

**Keywords:** functional neuroimaging, optical intrinsic signal, statistics, functional connectivity, multiple testing problem, random field theory

## Abstract

**Significance:**

Statistical inference in functional neuroimaging is complicated by the multiple testing problem and spatial autocorrelation. Common methods in functional magnetic resonance imaging to control the familywise error rate (FWER) include random field theory (RFT) and permutation testing. The ability of these methods to control the FWER in optical neuroimaging has not been evaluated.

**Aim:**

We attempt to control the FWER in optical intrinsic signal imaging resting-state functional connectivity using both RFT and permutation inference at a nominal value of 0.05. The FWER was derived using a mass empirical analysis of real data in which the null is known to be true.

**Approach:**

Data from normal mice were repeatedly divided into two groups, and differences between functional connectivity maps were calculated with pixel-wise t-tests. As the null hypothesis was always true, all positives were false positives.

**Results:**

Gaussian RFT resulted in a higher than expected FWER with either cluster-based (0.15) or pixel-based (0.62) methods. t-distribution RFT could achieve FWERs of 0.05 (cluster-based or pixel-based). Permutation inference always controlled the FWER.

**Conclusions:**

RFT can lead to highly inflated FWERs. Although t-distribution RFT can be accurate, it is sensitive to statistical assumptions. Permutation inference is robust to statistical errors and accurately controls the FWER.

## 1 Introduction

Statistical inference from neuroimaging data is complicated by the “multiple testing problem” in the setting of spatial autocorrelation. Comparing groups of images between experimental cohorts can involve tens of thousands of parallel pixel-wise statistical tests. Thus, even if the individual type I error rate for each individual pixel’s statistical test was effectively controlled at (for example) 0.05, we would expect hundreds of false positives pixels over the entire image. Controlling the familywise error rate (FWER: the likelihood of any false positive result occurring anywhere in the image) must account for the multiple statistical tests. However, neuroimaging data often have complicated spatial autocorrelation structures (arising from the point-spread function of the imaging system, processing choices such as spatial smoothing, and the underlying neuroanatomy), which makes it difficult to determine an appropriate statistical threshold for significance. Multiple methods have been developed to control the FWER^1^^,^^2^ for positron emission tomography (PET) and functional magnetic resonance imaging (fMRI). As statistical rigor has become a greater focus in the optical neuroimaging community,^3^ multiple groups have adapted such methods to functional near-infrared spectroscopy^4^^–^^6^ and widefield optical neuroimaging (WOI).^7^

Such methods are often demonstrated using a task in which a known response is expected or with two groups between which a difference is expected. The authors, then, demonstrate that their method can highlight the brain regions that are activated or that differ between groups. However, although this demonstration may illustrate adequate statistical power (i.e., limitation of type II error), it does not prove whether or not such methods control the FWER. Assessment of whether FWER control is adequate requires data in which the null hypothesis is known to be true. Our goal in the present manuscript is to determine whether algorithms designed to control the FWER perform as expected, i.e., does the actual FWER match the nominal value? In particular, we are interested in group analysis of resting-state functional connectivity (FC) data collected in mice using optical intrinsic signal (OIS) imaging, although the techniques described and the overall conclusions are generally applicable to other modalities or analysis methods.

Broadly, there are two ways of thinking about FWER control in neuroimaging. With pixel-wise inference, we are interested in finding a new threshold for significance that can be applied to individual pixels in the statistical image such that, if the null hypothesis were true, the probability that we would find pixels with values as extreme as (or more extreme than) this threshold would be equal to the target FWER (usually α=0.05). Alternatively, cluster-wise methods use a pixel-wise threshold to form clusters of potentially significant pixels and then attempt to find a threshold on the extent of spatially contiguous clusters to determine significance. In both cases, the significance threshold (and thus the p-value of an image feature) is based on the distribution of the maximum statistic: for pixel-wise inference, the probability that the highest peak in the statistical image will be at or above a value, and for cluster-wise inference, the probability that the largest cluster in the image will be larger than a certain extent. This distribution can be determined either non-parametrically, using permutation or boot-strapping methods, or parametrically by reference to an assumed null distribution. The most common parametric method used for neuroimaging is random field theory (RFT), which can be used with either pixel-wise or cluster-wise inference. RFT has recently been adapted to mouse optical neuroimaging as an open-source statistical processing toolbox for MATLAB.^7^ Once the probability distribution of the feature of interest is derived, individual features in the image (pixel heights or cluster sizes) can be assigned a p-value, which is the probability with which such a feature (or one more extreme) would appear if the null hypothesis were true. Although some publications^7^^,^^8^ include images of thresholded pixel-wise p-values after cluster analysis, properly, after assignment to clusters, the pixel-wise p-values have no statistical meaning, and each cluster (in its entirety) is assigned a single p-value.^9^ For any method, if the FWER is equal to the nominal value, then the test is exact. If the FWER is lower than expected, the test is conservative. If the FWER is higher than expected, then the p-values are falsely small and invalid.

The overall logic of the present study is inspired by Eklund et al.^10^ who analyzed fMRI statistical processing algorithms using a mass empirical analysis by resampling real data under the null. We use FC data taken from normal mice without any experimental intervention, mice are randomly and repeatedly divided into groups, and statistical tests of significance are applied. The null hypothesis of no differences between groups should always be true, and all pixels found to show statistically significant differences between groups will thus be false positives. Thus, the FWER of the testing procedures can be estimated. Using such methods, Eklund et al.^10^ found that the FWER for cluster-wise inference using RFT often substantially exceeded the nominal rate when implemented with many popular fMRI statistical toolboxes. We also demonstrate that the FWER with both pixel-wise and cluster-wise RFT in optical imaging is very sensitive to statistical assumptions and seemingly minor errors can lead to a substantially inflated FWERs (i.e., falsely low p-values). Finally, we also demonstrate how inference through permutation methods is able to control the FWER in a robust manner and is likely a better choice for statistical interpretation of optical neuroimaging data.

## 2 Methods

### 2.1 Optical Neuroimaging

**Fig. 1:**
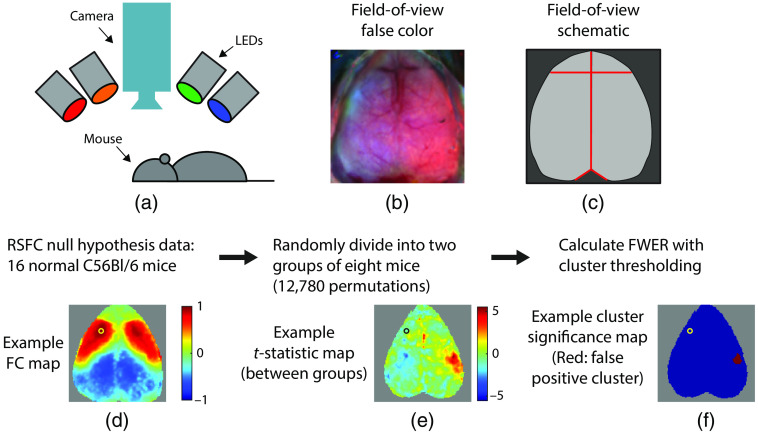
Schematic of the imaging system and methods. (a) OIS imaging system with four temporally multiplexed light-emitting diodes (LEDs) and an imaging camera positioned over the mouse. (b) False-color image demonstrating the field-of-view of the imaging system. The two hemispheres of the mouse brain are seen from above (left hemisphere on the left, anterior at the top of the page). (c) Schematic of the field-of-view demonstrating the segmented brain (light gray) and major landmarks (red). (d) Example resting-state functional connectivity map from an example mouse and seed (left motor cortex). (e) Example t map from a single permutation (left motor seed) demonstrating variance in the t statistic spatially under the null. (f) A false positive cluster is identified using Gaussian RFT (red pixels). As the null hypothesis is always true, the FWER is the percentage of times that any false positives occur.

All procedures were approved by the Institutional Animal Care and Use Committee of the Children’s Hospital of Philadelphia. Intact-skull cranial windows were placed on wild-type C57bl/6 mice (Jackson Laboratory, Bar Harbor, Maine). OIS imaging was performed using ketamine and xylazine anesthesia at ∼8.5 weeks of age (range: 6.9 to 12.1 weeks). OIS data were processed as previously described, including pixel-wise censoring, guided brain segmentation and atlas transformation, spectroscopy, and filtering to 0.01 to 0.1 Hz.^11^^–^^14^ All analyses were performed on normalized changes in total hemoglobin concentration after global signal regression. Images were acquired in 5-min scans with 5 to 6 scans per mouse (25 to 30 min of data). Data were concatenated across runs prior to functional connectivity analysis. Data consist of two-dimensional (2D) images of the superior mouse brain as viewed from above [Figs. 1(a)–1(c)].

Seed-based resting-state functional connectivity analysis was performed by selecting 14 seeds (7 per hemisphere) from major brain regions [i.e., motor, lateral somatosensory, medial somatosensory, visual, auditory, retrosplenial, and olfactory, Fig. 1(d)]. Relative to bregma, the seven seed locations in the left and left hemispheres were chosen symmetrically about midline (Table 1).

**Table 1:** Seed locations relative to bregma, as viewed from above.

Seed name	Medial/lateral	Anterior/posterior
Motor	1.68-mm lateral	2.27-mm anterior
Somatosensory (lateral)	2.85-mm lateral	0.24-mm anterior
Somatosensory (medial)	2.07-mm lateral	0.62-mm posterior
Retrosplenial	0.98-mm lateral	1.71-mm posterior
Visual	2.38-mm lateral	3.35-mm posterior
Olfactory	0.82-mm lateral	4.15-mm anterior
Auditory	3.40-mm lateral	1.63-mm posterior

Each seed trace was constructed by averaging the signal across a 5-pixel-radius (∼0.4-mm-radius) circle centered on the seed location. Correlation coefficients with each seed were converted to z-scores using Fisher’s transformation and a variance that accounted for temporal autocorrelation using “Bartlett’s method.”^15^

### 2.2 Empiric Determination of the FWER

Our goal is to determine the empiric rate by which false positives arise in between-group analysis of resting-state functional connectivity data in mice. We utilize a dataset of resting-state scans from 26 mice, considered as a whole and as a subset of the first 16 mice scanned. In both cases, mice were repeatedly divided into two equally sized groups. For the subset of 16 mice, there are $(\begin{array}{c} 16\\ 8 \end{array})=12,780$ total possible ways of randomly splitting the mice into two groups; all of these random permutations were performed and analyzed. For the entire dataset, there are $(\begin{array}{c} 26\\ 13 \end{array})=10,400,600$ possible permutations, so 10,000 were performed and analyzed.

As the segmented brain (and pixels masked for quality reasons) varied between mice, analyses were performed on all pixels where sufficient mice had evaluable data: for the N=16 cohort, analysis was performed at all pixels where at least 6 mice in each group had data, and for the N=26 cohort, the equivalent number was 11 mice in each group. For each permutation and for each seed, we assessed differences between the groups using pixel-wise two-sample Student’s t-tests to generate raw (uncorrected) p-values and t-statistics [Fig. 1(e)]. As the t-test assumes that the variables are normally distributed, it should be performed between the z-maps of each group. However, occasionally (as in Brier and Culver^7^), researchers perform the statistical test on the raw correlation coefficients, r. As the t-test is relatively robust to deviances from normality, we expected that this would be a minor concern, and we performed both analyses to test whether this difference affected the FWER.

From these t-maps, significant differences between groups were determined using the methods outlined below in detail, including (1) pixel-wise RFT, (2) cluster-wise RFT, (3) pixel-wise permutation analysis, and (4) cluster-wise permutation analysis [Fig. 1(f)]. For RFT, we varied the assumptions and algorithms to account for various possible methodologies as well as software bugs discovered in the open-source code used as a starting point. For all analyses, parameters were chosen with the goal of controlling the FWER at 0.05, as is typical in the scientific literature.

As all mice were normal mice, there should be no significant differences between groups. Any positive results were false positives. For any permutation, we are only interested in whether a false positive result is found and not whether multiple false positive pixels or clusters are found. Thus, for each analysis method and each seed location, the FWER is simply the number of permutations resulting in at least one false positive divided by the total number of permutations. The primary outcome of our analyses was whether the FWER approximated the nominal value of 0.05. All calculations were performed using MATLAB 2020a. The functional connectivity datasets used and the code necessary to calculate the FWER are provided online.

### 2.3 Random Field Theory

The use of RFT for task-based functional neuroimaging analysis has been covered in depth in many publications.^1^^,^^2^ Our goal here is to provide a brief summary of its key features, extend them to functional connectivity data, and focus on the various assumptions that affect this analysis for their application to (2D) widefield optical imaging. The current gold standard for using RFT in widefield optical imaging is the open-source software package published by Brier and Culver,^7^ which has been used in multiple publications.^8^^,^^16^^,^^17^ We adapted this software package to our data format. Modifications to its algorithms are described below, and at the request of reviewers, a list of all differences between our final algorithm and that of Brier and Culver^7^ with line references to their code is available in the Supplementary Material.

RFT assumes that, under null hypothesis conditions, test statistic images approximate a random field. We are interested in the excursion set, the expected number of clusters of pixels in a search region, V, with a test statistic that exceeds a threshold, T. For high enough T (such that we expect at most one cluster of pixels—without holes—exceeding this threshold), the expected number of clusters is given by [c.f., Eq. (7.11) in Ashby^2^ and Eq. (3.1) in Worsley et al.^18^]:

$$\mu =s\overset{2}{\underset{d=0}{\sum }}R_{d}(V)f_{d}(T),$$ (1)

where $R_{d}(V)$ is the number of d-dimensional “resels” (a neologism coined by Worsley et al.^19^ to mean resolution elements) in the search region and $f_{d}(T)$ is the expected value of the Euler characteristic in one resel of dimension d with the threshold T (methods for determining the search volume and resel count are discussed below). s is a correction factor to account for the sidedness of the statistical test. Usually, Eq. (1) is presented with s=1, which assumes that we are only concerned with the probability of finding a cluster of pixels where the value exceeds T. Although such a one-sided statistical test may be appropriate when attempting to determine which pixels are associated with a canonical hemodynamic response function in a task-based paradigm, here, we are interested in the differences between two group functional connectivity maps. As functional connectivity could increase or decrease in strength, we should allow our statistical test to be two-sided.^20^^,^^21^ As we are assuming that the null hypothesis is true everywhere, the distribution is symmetric about zero, and the likelihood of a cluster of pixels having values above T is the same as having values below −T. Thus, properly, for our analysis, s should be 2.

Often, in neuroimaging, the random field is assumed to be Gaussian, regardless of the origin of the test statistic (properly, using a probability integral transformation at each pixel). For Gaussian z-maps, the expected Euler characteristics are given as^18^

$$f_{0}(T)=\int_{T}^{\infty }\frac{1}{\sqrt{2\pi }}e^{-x^{2}/2}dx=1-\Phi (T),$$ (2)

$$f_{1}(T)=\frac{\sqrt{\ln(2)}}{\pi }e^{-T^{2}/2},\;\text{and}$$ (3)

$$f_{2}(T)=\frac{4\;\ln(2)}{{\left(2\pi \right)}^{3/2}}Te^{-T^{2}/2},$$ (4)

where Φ is the cumulative density function of the standard normal distribution. For t-fields (which are what result from the student’s t-test), the expected Euler characteristics are given as^18^^,^^22^

$$f_{0}(T)=\frac{\Gamma (\frac{\nu +1}{2})}{\sqrt{\nu \pi }\Gamma (\frac{\nu }{2})}\int_{T}^{\infty }{\left(1+\frac{x^{2}}{\nu }\right)}^{(\nu +1)/2}dx,$$ (5)

$$f_{1}(T)=\frac{\sqrt{\ln(2)}}{\pi }(1+\frac{T^{2}}{\nu }{)}^{-(\nu -1)/2},\;\mathrm{and}$$ (6)

$$f_{2}(T)=\frac{4\;\ln(2)}{{\left(2\pi \right)}^{3/2}}T(1+\frac{T^{2}}{\nu }{)}^{-(\nu -1)/2}.$$ (7)

Here, ν is the degrees of freedom in the t-test, which is the total number of subjects minus two, although as the brain segmentation is not equivalent across mice, the degrees of freedom is not invariant across the image. The gamma function, Γ, is an extension of the factorial function. It is worth noting that the equations for $f_{d}(T)$ of t-maps in Ashby^2^ are incorrect as the exponents are given as $-\frac{1}{2(\nu -1)}$.^2^^,^^23^

### 2.4 Pixel-Wise Analysis

When performing pixel-wise analysis, we are interested in a T threshold such that false positive pixels only appear with an FWER of α (e.g., 0.05). Thus, we solve Eq. (1) to find a T that results in μ=0.05. This answer is derived numerically. The pixel-wise p-value is the likelihood that a pixel of value T (or more extreme) would occur under null hypothesis conditions, which is the definition of μ in Eq. (1). It is worth noting that the equivalence between μ and p only holds for sufficiently high T.

### 2.5 Cluster-Wise Analysis

For cluster-wise analysis, we choose an initial T-threshold and then examine clusters of pixels above this threshold, with the goal of finding a value for cluster extent with which to control the FWER. For Gaussian RFT in the limit of high thresholds, the size of clusters follows a Poisson distribution,^2^^,^^24^ so the probability that a cluster has a size of least k pixels is given as

$$\beta =e^{-\lambda k},$$ (8)

where (in two dimensions) λ is given by [c.f., Eq. (12) in Friston et al.^24^ and Eq. (7.19) in Ashby^2^]:

$$\lambda =\frac{\Gamma (2)\mu }{sVf_{0}(T)}.$$ (9)

Here, μ is the expected number of clusters at threshold T, V is the number of pixels in the search volume, $f_{0}(T)$ is the single pixel likelihood of exceeding the threshold, and s is either 1 or 2 depending on test sidedness. It is worth noting that Eq. (8) only holds for Gaussian fields; although a version for t-fields has been derived,^25^ it is significantly more complex. In practice, Eq. (8) is often used for t-fields.^26^

We know that the expected number of clusters at a pixel threshold T is μ, given by Eq. (1). Then, the overall nominal FWER is one minus the probability that at least one of these clusters has a size over k pixels:

$$\alpha =1-e^{-\mu \beta }.$$ (10)

Recall that β is a function of k [Eq. (8)]. We then solve for k to determine the cluster size necessary to yield a nominal FWER of α [c.f., Eq. (15) in Friston et al.^24^ and Eq. (7.20) in Ashby^2^]:

$$k=\frac{1}{\lambda }\;\ln(-\frac{\mu }{\ln(1-\alpha )}).$$ (11)

It is worth noting that the choice of T (which determines μ and k) is arbitrary within the constraint that T should satisfy the original assumptions that justify the use of the Euler characteristic. In the limit of high T, any T can be chosen: lower pixel-wise thresholds result in a larger necessary cluster-size for significance. A typical choice (as in Brier and Culver^7^ and many fMRI studies) for the pixel-wise threshold is T=3.09, which corresponds to p=0.001, assuming the p-values are normally distributed. As the p-values are not necessarily distributed in a Gaussian manner, more properly, T needs to be determined based on the cumulative distribution function of the underlying statistical distribution. We also note that the correspondence between T=3.09 and p=0.001 assumes a one-tailed distribution.

Additionally, we found a crucial bug in the code provided by Brier and Culver^7^ wherein although a value of T=3.09 was chosen to calculate μ and k, in the definitive analysis, statistical images were thresholded at the Matlab default of p=0.05, equivalent to T=1.65 for a one-tailed normal distribution. We assess below the effect of this bug on the occurrence of false positives and thus the FWER.

Two common approximations for Eq. (9) (when assuming a normal distribution) are to neglect the lower-dimensional corrections to μ [i.e., $\mu =R_{2}(V)f_{2}(T)$ with $f_{2}(T)$ being defined by Eq. (4)] and to use a large T approximation to the normal cumulative density function [i.e., to $f_{0}(T)$; see Eq. (13) in Friston et al.^24^]:

$$1-\Phi (T)\approx \frac{e^{-T^{2}/2}}{T\sqrt{2\pi }}.$$ (12)

Then,

$$\lambda =\frac{2\;\ln(2)}{\pi }\frac{\Gamma (2)}{{\left(\mathrm{FWHM}\right)}^{2}}T^{2}.$$ (13)

This result is nearly identical to Eq. (8) in Brier and Culver,^7^ where they define

$$\lambda =\frac{4\;\ln(2)}{2\pi }\frac{\Gamma {\left(2\right)}^{2}}{{\left(\mathrm{FWHM}\right)}^{2}}T^{2}.$$ (14)

The difference between Eqs. (13) and (14) is an extra factor of Γ(2), which we believe was spuriously inserted.^27^

In summary, RFT yields Eq. (11), which gives a threshold of k pixels; clusters above this size are considered significant. To calculate the p-value to assign a given cluster, Eq. (10) is used.

We address in the Results (Sec. 3) whether these p-values are valid; here, it should immediately be noted that using a cluster-defining threshold of T=1.65 results in larger clusters than T=3.09. Thus, if μ and λ are defined using a threshold of T=3.09 but the cluster size is actually assessed for clusters defined using T=1.65 (as in Brier and Culver^7^), then the p-values will be invalid (i.e., the p-values will be falsely small and thus underestimate the actual rate at which such a cluster size is found under the null).

### 2.6 Search Volume and Resel Count

In some publications, the statistical search area is taken to be the entire field-of-view, including pixels outside of the brain segmentation.^7^^,^^24^^,^^28^ More properly, the search region should be restricted to the actual brain segmentation, which is the only region where differences between groups are of relevance. Furthermore, to account for the possibility that clusters touch the boundary, it is necessary to account for the number of resels in multiple dimensions [i.e., we should use the full sum in Eq. (1)]. Methods for numerically approximating the resel counts, R, were given by Worsley et al.^18^ and involve counting the number of resel faces and edges in the search region. It is important to note that these resel counts are approximations that are most accurate when the search region is convex. This assumption may not always be accurate in optical imaging in which individual pixels may be masked for quality control or the entire midline may be masked to divide the brain into two separate hemispheres. However, we are unaware of any published method to account for such search region topologies. So, we rely on the resel formulas from Worsley et al.^18^ as the best available method. In practice, we use the formulas of Worsley et al.^18^ to count the number of effective pixels in each dimension, d, and then divide by ${\mathrm{FWHM}}^{d}$ to convert to resels.

### 2.7 Empirical determination of FWHM

Following the methods of Xiong et al.^29^ and Keibel et al.,^30^ the full width at half maximum (FWHM) is estimated from the spatial roughness, Λ, as follows. If u(x,y) is an image, then (assuming unit pixel dimensions and N total pixels) the partial spatial derivatives are estimated as

$$[\begin{array}{c} u_{x}^{\prime }(x,y)\\ u_{y}^{\prime }(x,y) \end{array}]=[\begin{array}{c} u(x+1,y)-u(x,y)\\ u(x,y+1)-u(x,y) \end{array}].$$ (15)

The variance and covariance are then estimated as

$$V_{xx}=\frac{1}{N}\frac{\eta -2}{\eta -1}\underset{x,y}{\sum }{\left[u_{x}^{\prime }(x,y)-\langle u_{x}^{\prime }\rangle \right]}^{2},$$ (16)

$$V_{yy}=\frac{1}{N}\frac{\eta -2}{\eta -1}\underset{x,y}{\sum }{\left[u_{y}^{\prime }(x,y)-\langle u_{y}^{\prime }\rangle \right]}^{2},\;\text{and}$$ (17)

$$V_{xy}=\frac{1}{N}\frac{\eta -2}{\eta -1}\underset{x,y}{\sum }[u_{x}^{\prime }(x,y)-\langle u_{x}^{\prime }\rangle ][u_{y}^{\prime }(x,y)-\langle u_{y}^{\prime }\rangle ],$$ (18)

where η is the number of degrees of temporal freedom in the data, which we estimate using Bartlett’s method.^15^

The estimated variance-covariance matrix is

$$\Lambda =[\begin{array}{cc} V_{xx} & V_{xy}\\ V_{xy} & V_{yy} \end{array}].$$ (19)

The FWHM is then estimated as

$$\mathrm{FWHM}=\sqrt{\frac{4\;\ln(2)}{\sqrt{\left|\Lambda \right|}}}.$$ (20)

Although not described in the text of Brier and Culver,^7^ their method of calculating the FWHM can be found in the open-source code. The FWHM is estimated from the spatial autocorrelation of the image. The autocorrelation is calculated along the x- and y-dimensions, and the FWHM is calculated as the width of the autocorrelation curve at a value of $1/\sqrt{2}$. We are not aware of a justification for this method being published in the prior literature.^27^ It is worth noting that there is a small bug in the code such that the origin is calculated twice and the FWHM is spuriously one pixel too large.

More properly, the roughness should be calculated on the statistical image (i.e., the t-map) rather than the hemodynamic data, or if it is assumed that the statistical map might have signal, the roughness should be calculated from the map of residuals, which are assumed to have the same structure as the statistical map without the signal. With our data, there is no signal, as the null hypothesis should always be true. So, we expect either method to yield similar results.

We compare the FWHM derived from both the spatial roughness and spatial autocorrelation methods. To provide similarity with Brier and Culver,^7^ we calculated both on the hemodynamic data prior to functional connectivity correlation analysis; this analysis was performed on all mice and averaged across frames and then across mice. We also calculated FWHM using both methods on the t-maps and residuals; for this analysis, we selected 100 random possible permutations and calculated the mean and standard deviation across permutations and seeds. For analyzing the statistical residuals, this was performed with the same 100 permutations as above with averaging across seeds and mice.

### 2.8 Random Field Theory Summary

We detailed multiple methods to use RFT to attempt to control the FWER and multiple parameters in the model that can be adjusted. To summarize, the following is a list of the options:

1. Expected Euler characteristic formulas: Gaussian z-distribution versus student’s t-distribution,
2. One-sided versus two-sided statistical tests,
3. Euler characteristic dimensionality: Use only 2D resels or use the “unified” sum across dimensions,
4. The full definition of λ [Eq. (9)] or the simplified definition [Eq. (13)],
5. The value of the FWHM, and
6. The statistical search area: the full 2D area versus the segmented brain.

In addition to the above, we also tested the effect of the thresholding bug in the code of Brier and Culver.^7^ As all of these choices offer many possible statistical methods, we only show the results below for some relevant combinations. The code provided online can be used to generate the FWER for other combinations as desired by the reader.

### 2.9 Permutation Inference

With permutation inference, rather than assuming a form for the distribution of the maximal statistic from RFT, the distribution is estimated from the data itself. For each possible permutation of the data, the maximal value of the test statistic $T_{\max}$ is calculated across the image. Over all M images, we now have an empirical distribution of $T_{\max}$. To perform pixel-wise analysis, with a nominal FWER of α, all of the M values are sorted from smallest to largest. Then c=⌊αM⌋+1 is defined. The critical value for the test statistic is then the c’th largest value of $T_{\max}$ in our distribution, $T_{c}$. We reject the null hypothesis for any pixels with $T>T_{c}$.^31^^,^^32^ For a two-tailed test, the same analysis is performed with ${\left|T\right|}_{\max}$. The p-value for any pixel is simply calculated by comparing its t-value to the empirically derived cumulative density function of the maximum statistic.

A similar analysis can be performed for cluster-wise analysis. Again, we have to choose a predetermined T threshold with which to define the clusters. For consistency with the RFT analysis, we use the same values as above, derived from the values of T expected to correspond to p=0.001 (from one-sided distributions). Then, for each permutation, we calculate the largest cluster size found above that threshold. Again, we sort these sizes from smallest to largest and determine the c’th largest value in this distribution using the same algorithm as above. We then reject the null hypothesis for any clusters exceeding this threshold.^32^ As above, the p-value for a cluster is calculated by comparing its extent to the empirically derived cumulative density function of the maximum cluster size. It is worth noting that, with permutation inference, p-values are always quantized in increments of one over the number of permutations evaluated.

Because our gold standard for determining the FWER is to perform a comprehensive permutation analysis, using the above algorithm on the entire dataset would by definition control the FWER at exactly the nominal level. So, instead, we ask how well performing permutation inference on a subset of the data generalizes to the entire dataset. We performed the analysis with all mice in the *N* = 16 and *N* = 26 groups, but only examined 1000 randomly selected permutations to derive pixel-wise and cluster-wise significance thresholds and then applied these to the entire set of permutations.

## 3 Results

### 3.1 Determining the FWHM

**Fig. 2:**
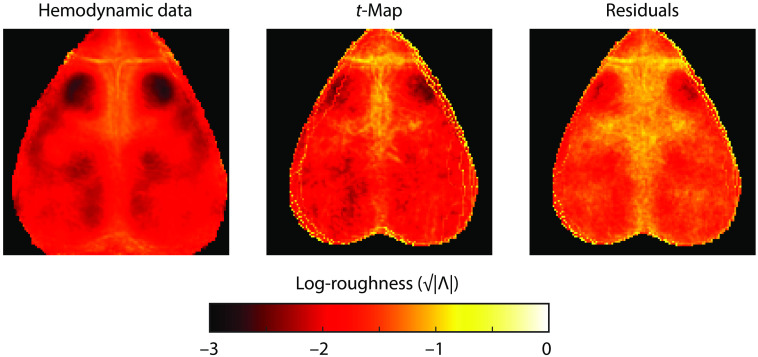
Estimated spatial roughness (on a log-scale), $\sqrt{\left|\Lambda \right|}$ from the spatial partial derivatives of the hemodynamic data, t-maps, and residuals. The average was taken across mice, permutations, and seeds.

Before examining the FWER, we attend preliminarily to calculating the FWHM, a key component of RFT. First, we used the partial spatial derivatives to estimate roughness and then the FWHM. Using this method, all possible input images gave roughly similar FWHM. Determining the FWHM using the t-maps gave an FWHM of 8.9 pixels (approximately equal to 0.70 mm). Using the residuals gave an FWHM of 6.8 pixels (0.53 mm). Using the normalized data (as in Brier and Culver^7^), the estimated FWHM was 8.1 pixels (0.63 mm). The roughness, $\sqrt{\left|\Lambda \right|}$, did vary over the field-of-view (Fig. 2), with higher roughness along the venous sinuses (sagittally and between the cerebrum and olfactory bulb) and near the edges of the field-of-view. Roughness was lower in the middle of large cerebral regions where spatial autocorrelation was likely strongly driven by local functional connectivity (particularly motor cortex, but also somatosensory and retrosplenial).

Next, we calculated the FWHM using the spatial autocorrelation (as in the code of Brier and Culver^7^). This method was more variable. Using the hemodynamic data, the FWHM was larger at 12.2 (0.95 mm), which was slightly larger than with the spatial roughness method. However, when analyzing the t-maps and residuals, the FWHM was much higher, at 30.5 (2.38 mm) and 31.3 pixels (2.44 mm), respectively.

Overall, we believe that using the spatial partial derivatives on the t-maps is probably the most accurate method to estimate the FWHM of the data [FWHM 9 pixels, (0.70 mm)]. However, to demonstrate how the FWHM affects RFT analysis, we performed the mass permutation analysis with a range of assumed FWHMs from 4 to 15 pixels (0.31 to 1.17 mm).

### 3.2 Familywise Error Rate

We attempted to control the FWER using a variety of statistical methods, using a range of reasonable parameters. The FWER was determined using a mass empirical analysis. Recall that the nomimal FWER was always 0.05. The mass permutation analysis was always two-tailed. References below to “one-sided” and “two-sided” refer to the forms of the equations used to generate the significance threshold, which was then always applied to both positive and negative t-values.

#### 3.2.1 Gaussian random field theory

First, we assessed cluster-based Gaussian RFT. We started with the algorithms and methodology from Brier and Culver,^7^ which includes (1) using the full 128 pixel by 128 pixel image as the statistical search space, (2) using the simplified approximation of Eq. (13) for the expected number of pixels in a cluster, and (3) including the bug that thresholds pixels at p=0.05 despite calculating the cluster size threshold with p=0.001. We started with an estimated FWHM of 12 pixels. These choices result in a cluster size threshold of 63 pixels for N=16 and 79 pixels for N=26. Performing statistical tests on the r-maps, these algorithmic choices lead to an extremely high FWER of 0.92 for N=16 and 0.83 for N=26 (ranges: 0.88 to 0.96 and 0.80 to 0.86, respectively, where the range is across the seed locations). To rephrase this result, over 80% of experiments (with the exact number dependent on sample size) when assessed with this algorithm show a “statistically significant” cluster of pixels as differing between the groups when no true difference exists, which is much higher than the nominal (and typically accepted) rate of 5%. The FWER did not substantially change when performing the statistical tests on the z-maps, with a median FWER of 0.92 (range: 0.89 to 0.96) for N=16 and 0.84 (range: 0.81 to 0.88) for N=26. As there were no major differences between performing statistical tests using the r- or z-maps, all subsequent results use the z-maps.

This high FWER is primarily due to the bug that incorrectly creates larger clusters. However, other statistical errors in the open-source algorithm have important and counter-acting effects. Incorrectly using one-sided equations for a question that involves two-sided inference results in a larger than expected FWER. Conversely, artificially increasing the field-of-view to include regions of the image outside of the brain segmentation (and similarly failing to account for the boundary’s effect on the Euler characteristic) artificially depresses the FWER. Fixing all of these errors (and continuing to use an FWHM of 12 pixels) results in an FWER of 0.28 (range: 0.25 to 0.32) for N=16 and 0.081 (range: 0.057 to 0.091) for N=26.

**Fig. 3:**
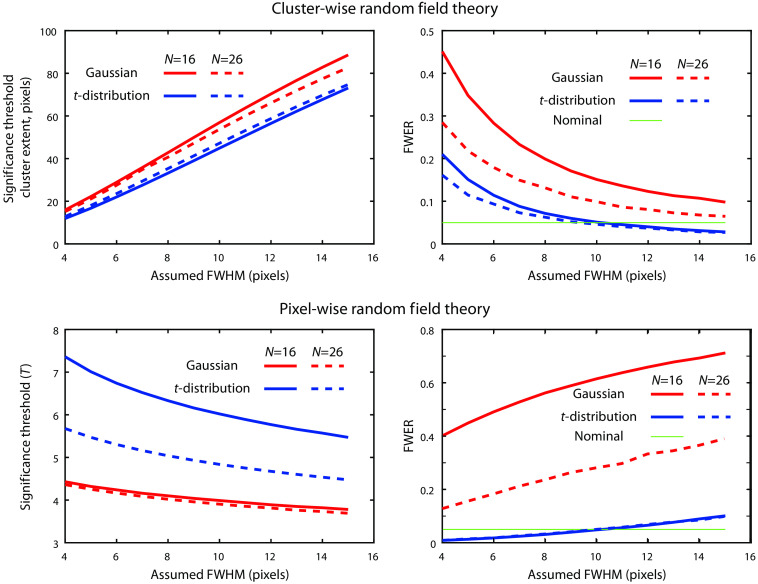
Numerical comparison of methods to control the FWER. The FWER is shown as well as the threshold for determining significance (either a statistical threshold T, or a cluster size threshold k). This analysis uses two-sided equations, the full version of Eq. (9), the full dimensionality for Eq. (1), and the segmented brain.

The performance of Gaussian cluster-based RFT inference using equations corrected for two-tailed inference and the limited search area are shown in Fig. 3 for a variety of FWHMs and both sample sizes. It is clear that the results of Gaussian RFT are very sensitive to the FWHM used (and we have previously seen how FWHM is itself very sensitive to processing choices). Across all FWHM choices, the FWERs are substantially higher than the nominal value (which means that any p-values are invalid). Pixel-wise Gaussian RFT also resulted in spuriously high FWERs (Fig. 3) at all reasonable FWHM choices. It is interesting to note that changing the FWHM has opposite effects on cluster-wise and pixel-wise RFT. With cluster-wise RFT, a larger FWHM (assumed smoother data) means that clusters are expected to be broader. Thus, the size threshold for significance is increased and the FWER falls. However, for pixel-wise RFT, a larger FWHM means that the multiple testing problem is less severe; the T threshold for significance is lower, and the FWER is higher. It is also interesting to note the effect of sample size. The sample size has minimal effect on the significance threshold for either cluster- or pixel-based inference (as the Euler characteristics of Gaussian fields are independent of sample size). However, the larger sample size universally results in a much lower (more appropriate) FWER. This result indicates that the distribution of noise in the data (unlike a Gaussian) is dependent on sample size—as the sample size increases, the data more closely approximates a Gaussian distribution.

**Fig. 4:**
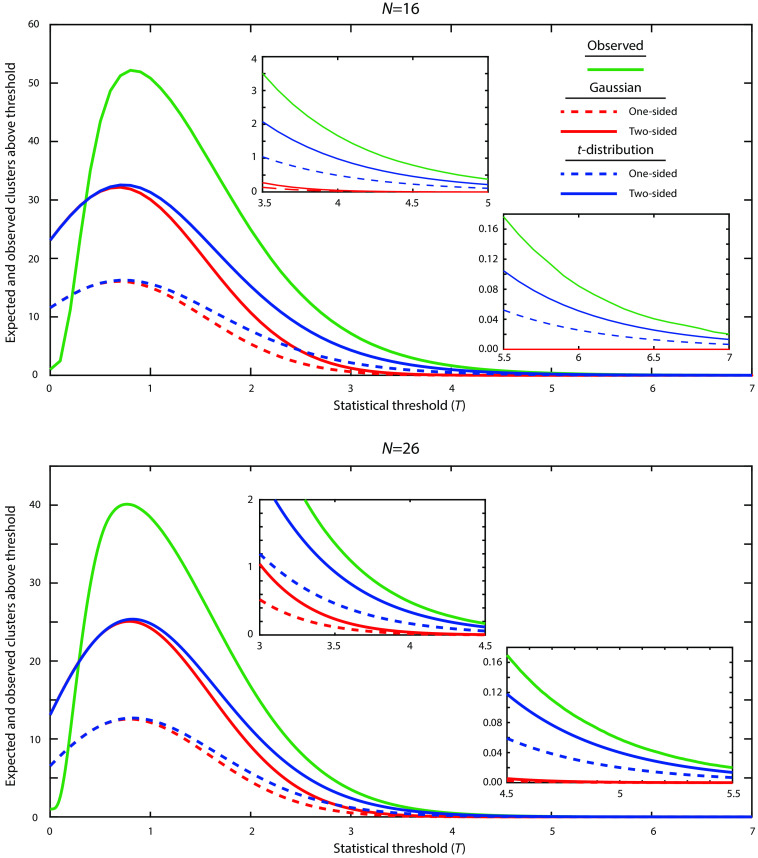
Observed versus expected number of clusters above a threshold |T| for both N=16 and N=26 datasets. The observed number of clusters was determined empirically (green). The expected number of clusters in a Gaussian random field is shown in red for both one-sided (dashed) and two-sided (solid) analysis. The expected number of clusters in a t-field is shown in blue for both one-sided (dashed) and two-sided (solid) analysis. The insets show the same data zoomed in where the observed value crosses 1 and 0.05.

To assess the failure of Gaussian RFT, we plotted the observed number of clusters as a function of |T| as well as the expected number of clusters as predicted from RFT [Eq. (1) using Eq. (4)]. Recall that, for pixel-wise RFT, we numerically solve the Euler characteristic equations to predict when the observed line should cross below 0.05. For cluster-wise RFT, we choose an initial threshold for T, which should be near where this line crosses 1. We see that the observed behavior has a much longer “tail” at a high T than would be expected for a Gaussian distribution (Fig. 4). As the T-threshold for cluster analysis should be chosen such that on average less than one cluster should appear per statistical image, this assumption is clearly violated at T=3.09 (p=0.001 assuming a Gaussian distribution), where the observed number of clusters averages 6.2 per statistical image for N=16. The cluster distribution (at high T) more closely follows the expected number of clusters for a two-sided t-distribution [Eq. (1) using Eq. (7)], as should be expected. However, even the two-tailed t-distribution still underestimates the number of observed clusters for each T. The t-distribution is reasonably close to the observed distribution when the expected number of clusters drops below 1 (i.e., where an appropriate cluster-threshold would be) and below 0.05 (i.e., the pixel-wise t-value threshold for significance). With the larger sample size (and increased degrees of freedom), the observed distribution and the predicted t-distribution more closely approximate a Gaussian distribution. However, the t-distribution and especially the Gaussian continue to underestimate the number of clusters in the empirical distribution at all thresholds.

**Fig. 5:**
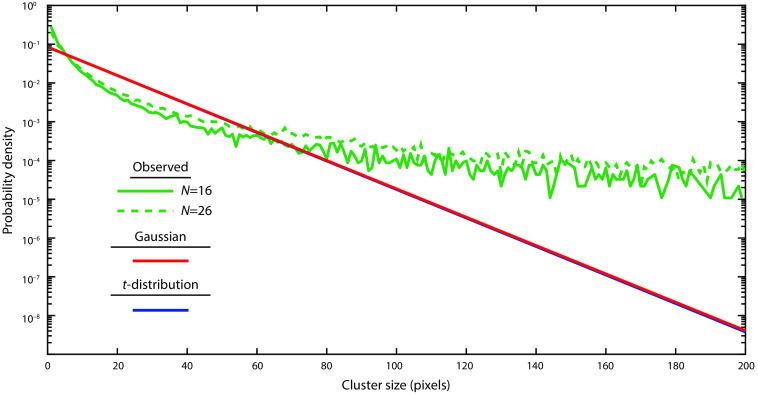
Expected and observed probability density functions for cluster size for both N=16 and N=26 datasets. The observed cluster sizes were determined empirically (green). The expected sizes were Poisson distributions with the expected mean value of μ as calculated for both Gaussian and t distributions (red and blue, respectively). It is worth noting that the two lines derived from RFT substantially overlap, so the blue of the t distribution is partially hidden.

In addition, the distribution of cluster sizes as predicted from Gaussian and t RFT (i.e., a Poisson distribution) poorly corresponds to the observed data (Fig. 5). The observed data show a sharper peak of very small clusters as well as a long tail of very large clusters. Thus, even when adjusting the assumed statistical distribution, RFT still does a poor job of predicting the structure of observed data. This result holds for both sample sizes.

#### 3.2.2 t-distribution random field theory

Based on Fig. 4, we expect that using the Euler characteristic equations for t-fields would yield more appropriate FWERs. For cluster-wise analysis, the use of a threshold of p=0.001 is associated with T=3.79. For all FWHM choices, the FWER for t-distribution RFT is lower than for Gaussian distribution. The results at a FWHM of 10 pixels approximated the nominal value of 0.05 (Fig. 3) for both N=16 and N=26. As expected, the use of pixel-wise t-distribution RFT results in substantially higher T thresholds for significance and lower FWERs, with this analysis being overly conservative at lower assumed FWHMs. Again, the nominal value of 0.05 is approximately met when using an FWHM of 10 pixels (Fig. 3).

### 3.3 Permutation Analysis

Permutation inference was performed using thresholds derived from a random subset of 1000 permutations that were then applied to the complete set of all possible permutations. As with RFT, for cluster-wise inference, we again have to pick an initial clustering threshold. We used both T=3.09 and T=3.79 to match the RFT analyses. Using the lower cluster-defining threshold results in a very large cluster size for significance of 171.2±37.6  pixels (the standard deviation is across seeds). The higher threshold results in a cluster size for significance of 48.4±7.0  pixels, which is similar to t-distribution with an FWHM of 10 pixels. With either threshold, cluster-wise permutation inference is able to control the FWER at ∼0.05 (0.042 and 0.045, respectively, for N=16 and 0.051 and 0.047, respectively, for N=26). Similarly, pixel-wise permutation inference results in a threshold for significance of T=6.18±0.13 (again similar to that from t-distribution RFT) with an FWER of 0.052 for both N=16 and N=26. Recall that this analysis was performed with 1000 permutations; increasing the number of permutations allowed would increase the accuracy of achieving an FWER of 0.05.

### 3.4 Example p-values

**Fig. 6:**
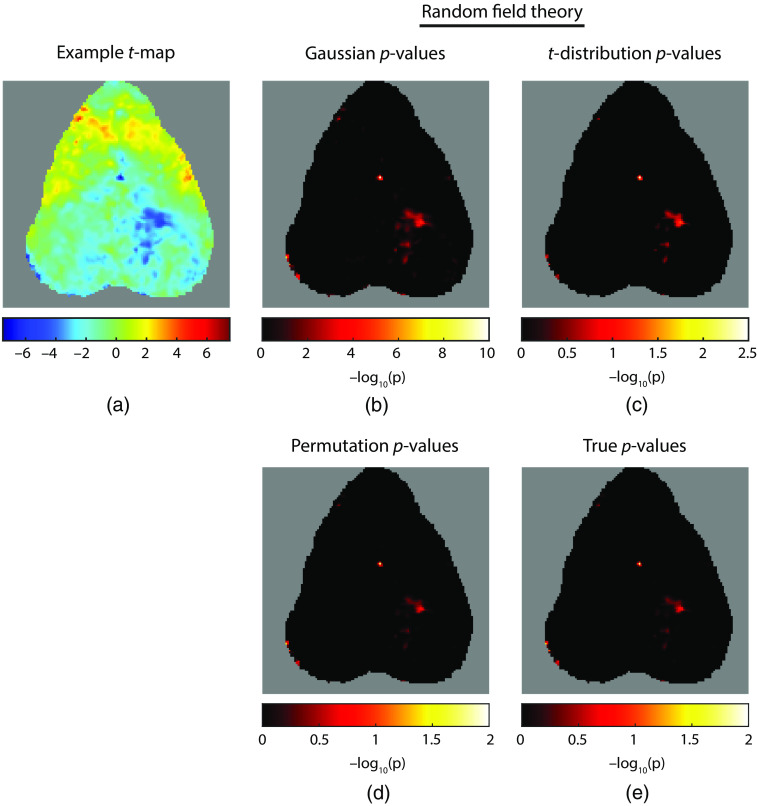
Calculation of p-values from an example random permutation of null hypothesis data (N=16). (a) Example t-map. p-values (displayed as $-\log_{10}(p)$ to better highlight small p-values) for individual pixels calculated with (b) Gaussian RFT, (c) t-distribution RFT, and (d) permutation inference. (e) p-values derived from the observed cumulative density function of the image maximum statistic. (It is worth noting the different scales across images.) Gaussian RFT greatly exaggerates the significance of pixels. t-distribution RFT also exaggerates significance, but to a lesser extent.

As p-values may be more familiar than the FWER, we can demonstrate how an inflated FWER results in falsely low p-values. We selected an example permutation (from the N=16 data characterized by a number of regions of high and low t-statistics [Fig. 6(a)]. For RFT analysis, we used an FWHM of 10 pixels and the full equations for μ, λ, and the field-of-view. We can see that both Gaussian distribution and t-distribution pixel-wise RFT [Figs. 6(b) and 6(c)], respectively) yield lower p-values than the either the permutation analysis [on the subset of 1000 permutations, Fig. 6(d)] or the gold standard [Fig. 6(e)]—in the case of Gaussian RFT, the p-values are strikingly small. For example, focusing on the pixel with the largest deviation from zero (which is a pixel near the center of the field-of-view with a value of t=−7.30, this pixel is assigned a p-value of $p=3.4\times 10^{-10}$ by Gaussian RFT and $p=4.5\times 10^{-3}$ by t-distribution RFT. Yet this pixel’s p-value via permutation inference is 0.018, and the true p-value based on the observed distribution is 0.0174.

**Fig. 7:**
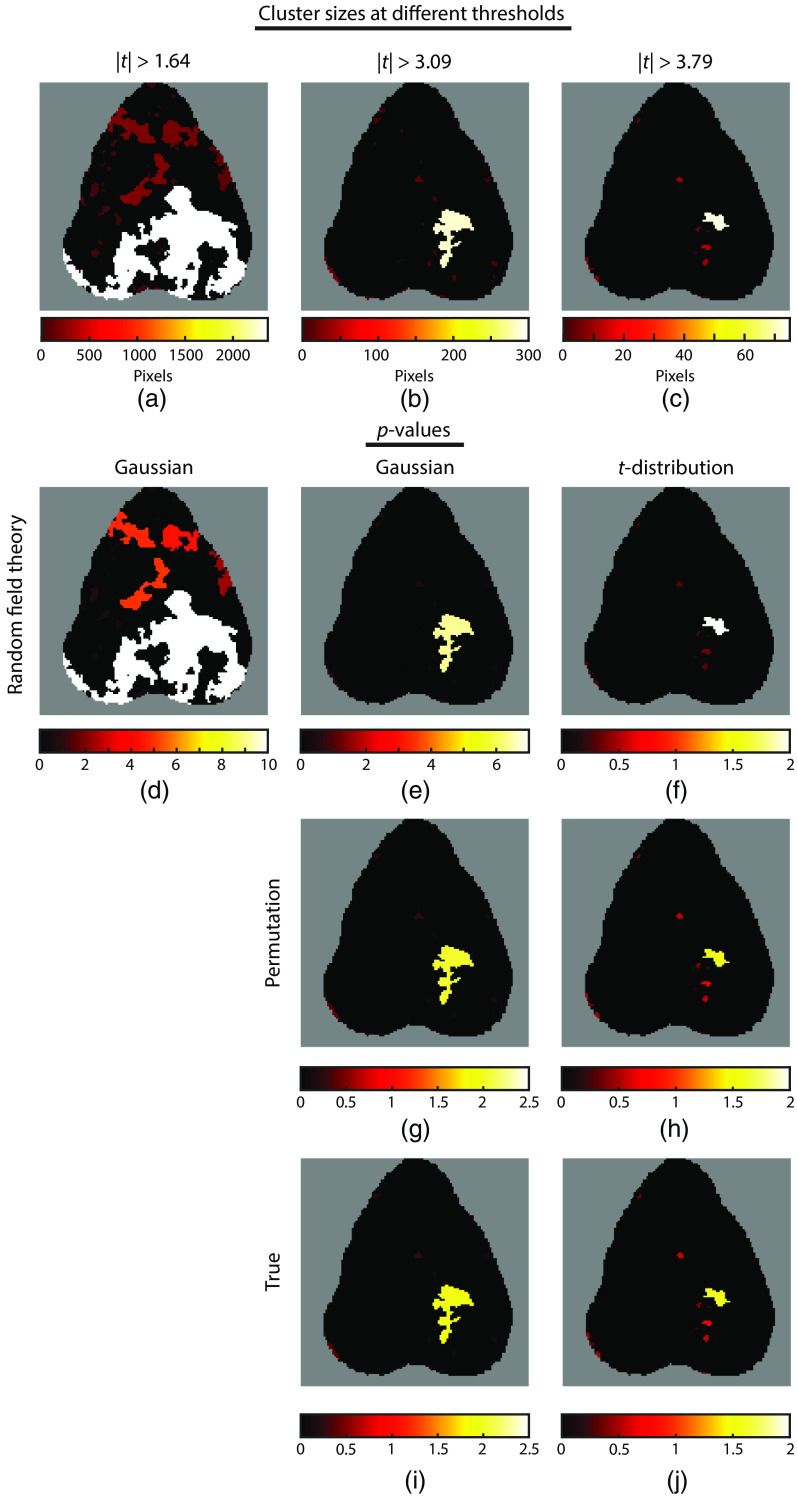
Calculation of p-values using cluster-wise inference; clusters are derived from the t-map in Fig. 6(a). Cluster sizes for different pixel thresholds: (a) the spuriously low threshold in Brier and Culver;^7^ (b) a typical threshold for Gaussian RFT; and (c) an equivalent threshold for t-distribution RFT. (d) p-values for the cluster sizes in (a) based on Gaussian RFT (which assumes pixels were actually tresholded at |t|>3.09. The p-values are extremely low, with a floor enforced at $p=10^{-10}$. (e) p-values based on Gaussian RFT for the clusters in (b). (f) p-values from t-distribution RFT for the clusters in (c). (g) and (h) p-values based on permutation inference for the clusters in (b) and (c), respectively. (i) and (j) Cluster p-values based on the observed distribution for the data in (b) and (c), respectively. Again, we see that Gaussian RFT greatly exaggerates significance.

Similar issues arise with cluster-based RFT. First, we note that the effect of the bug in the cluster-defining threshold in Brier and Culver,^7^ which defines clusters as those with |t|>1.64, is to create extremely large clusters covering much of the brain [Fig. 7(a)] compared with those properly defined as |t|>3.09 for Gaussian and |t|>3.79 for t-distribution RFT [Figs. 7(b) and 7(c), respectively]. When compared with the parametric distribution for Gaussian RFT (which assumes the correct threshold), the p-values for the large cluster are incredibly small. The p-value for the largest cluster (encompassing much of the posterior brain) is smaller than the minimum variable size in MATLAB (i.e., below about $2\times 10^{-308}$), so we assign it a value of $p=10^{-10}$ for display purposes [Fig. 7(d)]. With proper thresholding, the largest cluster is located in the right parietal lobe (being slightly larger with the lower threshold used for Gaussian RFT). Gaussian RFT still assigns this cluster a very low p-value of $p=4.17\times 10^{-7}$ [Fig. 7(e)], whereas permutation analysis assigns it a p-value of $p=9\;\times 10^{-3}$ [Fig. 7(g)], and the observed distribution assigns it a p-value of 0.0118 [Fig. 7(i)]. With t-distribution RFT, the p-values are more reasonable but still falsely low: p=0.010 for the largest cluster [Fig. 7(f)] compared with p=0.0260 with permutation inference [Fig. 7(h)] and p=0.0254 with the observed distribution [Fig. 7(j)].

## 4 Discussion

We performed a mass empirical permutation analysis to assess the FWER for statistical analysis of inter-group differences in optical resting-state functional connectivity. We found that the FWER for Gaussian RFT often greatly exceeded the nominal value. This finding means that the p-values reported by Gaussian RFT are invalid and the actual rate at which such features arise under the null is much higher than the p-value would naïvely suggest. Although it is possible to tweak the parameters of t-distribution RFT sufficiently to obtain an FWER near the nominal value, this result is highly sensitive to the statistical assumptions that may not generalize to other datasets. The appeal of RFT is that the complexities of the multiple testing problem can be condensed to a small set of equations; however, these advanced methods are not useful if we do not rigorously ensure that they behave as expected. The problems with RFT have been documented with fMRI inference;^10^ it was found that a combination of software bugs and improper statistical assumptions resulted in inflated FWERs for task-based analyses (we are not aware of prior work specifically addressing functional connectivity analysis). Improper usage of one-sided equations for two-sided questions has also led to FWER inflation in fMRI.^20^^,^^21^ Our analysis found similar problems.

One of the dangers of statistical toolboxes is that they can serve as a black box wherein data is entered and results of statistical significance are output, often without an assessment of whether the assumptions of the toolbox match the data at hand. The dissemination of analysis methods through open-source code is important for rigor, standardization of analysis, and reproducibility. Although it is possible to process optical data using existing fMRI analysis programs, such methods should be used with caution. The statistical properties of optical data can differ substantially from fMRI data,^15^ and it is unclear if results from fMRI-specific programs would be valid, absent an analysis comparable to the present paper. Thus, we applaud the efforts of Brier and Culver^7^ for developing the first freely available analysis software for widefield optical imaging in mice. Their work is a major step in adapting methods to optical neuroimaging, and it served as the foundation for our present analysis. Although we found bugs in their code, which dramatically inflate the FWER, we have alerted those authors to the errors^27^ and some of these errors were addressed in updated code, released during revision of the present manuscript. The code for the present analysis is available through Code Ocean, and although it is not a plug-and-play toolbox, the algorithms encapsulated in the code could be adapted for other’s use.

However, the poor performance of RFT in controlling the FWER is not entirely attributable to bugs in code. Fundamentally, it is important to rigorously examine the statistical assumptions in analysis and ensure that the observed data (and the questions being asked) satisfy those criteria. Importantly, in our present analysis the data did not follow a Gaussian distribution and the underlying scientific question called for a two-tailed analysis. Using one-sided Gaussian RFT equations originally derived for task-based analysis of PET and fMRI images resulted in severely inflated FWERs. Although using a two-sided t-distribution as the basis for RFT analysis did result in acceptable FWERs if the FWHM was carefully tuned, graphs of observed vs. expected cluster numbers and sizes clearly demonstrate that RFT does not fully capture the nature of the experimental data. As the experimental data is derived from a t-test, the resulting variance under the null is highly dependent on the degrees of freedom. As the sample sized increased, the data more closely approximated a Gaussian distribution and Gaussian RFT resulted in lower FWERs. However, the p-values for Gaussian RFT remained invalid within a range of reasonable FWHMs. The exact FWER obtained depended on the method by which resels were calculated (i.e., the treatment of the statistical search space, dimensionality, and calculation of the FWHM); proper treatments for all of these factors are themselves complicated or unclear, and they may vary significantly between datasets. As previously mentioned, the unified correction for the Euler characteristic proposed by Worsley et al.^18^ is only an approximation for a convex search space. Brain segmentations with concavities (e.g., masking of the midline) or holes (e.g., from pixel-wise censoring of low quality data) could result in unexpected effects on the Euler characteristic. Similarly, we have seen how different methods to calculate the FWHM can result in large differences in the calculated FWHM and the resulting FWER.

It is important to note that the FWHM metric in RFT is dependent on both the imaging system and the experimental design. The FWHMs calculated for this dataset are larger than the theoretical point-spread function of either the hemodynamic^33^ or fluorescence^34^ imaging system. This is partly due to widefield illumination and an intact skull. But also, spatial autocorrelation in the data is driven by underlying neurologic activity. Nearby cortical regions can be expected to have similar functional connectivity profiles and thus have correlated image noise. Furthermore, although the FWHM of the image noise is affected by the FWHM of any spatial smoothing applied to the data, the two cannot be assumed to be identical. Thus, the optimal FWHM of 10 pixels for t-distribution RFT found for the current dataset should not naïvely be applied to any future data from the same system let alone another hemodynamic imaging system or fluorescence data.

In contrast to RFT, we found that permutation inference, either pixel-wise or cluster-wise, was able to control the FWER as predicted. Permutation inference is not immune to algorithmic errors. For example, it is important to ensure that the empiric maximal distribution is determined in a two-tailed manner when appropriate. Although cluster-wise analysis is sensitive to the initial pixel threshold chosen, the cluster size threshold for significance varies such that the FWER can still be controlled even for low pixel thresholds. To a certain extent, our permutation results are biased as the gold standard was itself a permutation test. However, our results demonstrate the power and simplicity possible with permutation inference, and the 1000 permutations used to derive the thresholds are on the low end of recommended ranges for neuroimaging experiments.^32^ Overall, permutation methods are more robust to errors and do not rely on assumptions about the distribution of the data. Furthermore, permutation methods can be easily extended to other optical imaging methods (e.g., widefield fluorescence imaging, near-infrared spectroscopy, or diffuse optical tomography). As long as the permutation scheme reflects the underlying null hypothesis and the maximal statistic is defined properly, permutation methods are less dependent on the idiosyncrasies of each imaging system or contrast. Thus, we would recommend permutation inference as the preferred method for optical neuroimaging data.

It is important to note that we tested the ability to control FWER, that is the type I error. In data without any true differences between groups, we cannot assess power or the type II error rate (the rate at which the null hypothesis is falsely confirmed). With cluster analysis (either using RFT or permutation), there is additionally a balance between the FWER, power, and desire to detect spatially localized changes. Low cluster-defining thresholds will result in larger cluster-extent thresholds for significance (at a constant FWER). It is important to understand this interaction when choosing and then tuning a statistical method. In addition to the pixel-wise and cluster-extent method considered here, others have proposed more advanced methods for inference based on peak height.^35^ However, such methods often assume a Gaussian random field, which, as we have seen, is often a poor approximation to the data.

Furthermore, it is important to note that cluster-based inference methods are affected by inhomogeneities in image smoothness (i.e., nonstationarity). Areas of higher smoothness are more likely to have larger clusters of pixels exceeding a threshold. This problem is common to RFT and permutation methods. It is unclear to what extent inference in WOI is affected by nonstationarity. More advanced methods can attempt to account for differences in spatial roughness, but examining them is outside the scope of the present work.

Overall, our results demonstrate that statistical methods for between-group comparison can often result in FWERs that vastly exceed the desired nominal value. In particular, RFT methods are prone to algorithmic errors and are sensitive to statistical assumptions. RFT can result in highly inflated FWERs, which means that the resulting p-values and statistical significance should be interpreted with extreme caution when using these methods. Permutation inference, on the other hand, is not dependent on as many statistical assumptions and can more robustly control the FWER, resulting in p-values that accurately reflect the rate at which features arise under the null. When choosing a statistical method for a group experiment, it is essential to ensure that the FWER is controlled as expected (ideally with an analysis of null hypothesis data); otherwise, the results could be unexpectedly corrupted by false positive results.

## Code, Data, and Materials Availability

The datasets used in the analysis and the code used to generate the data and figures are available for use on Code Ocean: https://doi.org/10.24433/CO.7947046.v1.

## References

[1] Nichols T. Hayasaka S., “Controlling the family-wise error rate in functional neuroimaging: a comparative review,” Stat. Methods Med. Res. 12, 419–446 (2003). 10.1191/0962280203sm341ra 14599004

[2] Ashby F., Statistical Analysis of fMRI Data, 2nd ed., Massachusetts Institute of Technology (2019).

[3] Yucel M. et al., “Best practices for fNIRS publications,” Neurophotonics 8, 012101 (2021). 10.1117/1.NPh.8.1.012101 33442557 PMC7793571

[4] Ye J. et al., “NIRS-SPM: statistical parametric mapping for near-infrared spectroscopy,” NeuroImage 44, 428–447 (2009). NEIMEF 1053-8119 10.1016/j.neuroimage.2008.08.036 18848897

[r5] Tak S. Ye J., “Statistical analysis of fNIRS data: a comprehensive review,” NeuroImage 85, 72–91 (2014). NEIMEF 1053-8119 10.1016/j.neuroimage.2013.06.016 23774396

[6] Hassanpour M. et al., “Statistical analysis of high density diffuse optical tomography,” NeuroImage 85, 104–116 (2014). NEIMEF 1053-8119 10.1016/j.neuroimage.2013.05.105 23732886 PMC4097403

[7] Brier L. Culver J., “An open source statistical and data processing toolbox for wide-field optical imaging in mice,” BioRxiv (2021). 10.1117/1.NPh.10.1.016601 PMC9976616 36874217

[8] Rahn R. et al., “Functional connectivity of the developing mouse cortex,” Cereb. Cortex 32, 1755–1768 (2022). 53OPAV 1047-3211 10.1093/cercor/bhab312 34498678 PMC9016285

[9] Woo C. Krishnan A. Wager T., “Cluster-extent based thresholding in fMRI analyses: Pitfalls and recommendations,” Neuroimage 91, 412–419 (2014). NEIMEF 1053-8119 10.1016/j.neuroimage.2013.12.058 24412399 PMC4214144

[10] Eklund A. Nichols T. Knutsson H., “Cluster failure: why fMRI inferences for spatial extent have inflated false-positive rates,” Proc. Natl. Acad. Sci. U. S. A. 113, 7900–7905 (2016). 10.1073/pnas.1602413113 27357684 PMC4948312

[11] White B. R. et al. “Imaging of functional connectivity in the mouse brain,” PLoS One 6, e16322 (2011). POLNCL 1932-6203 10.1371/journal.pone.0016322 21283729 PMC3024435

[r12] White B. et al., “Brain segmentation, spatial censoring, and averaging techniques for optical functional connectivity imaging in mice,” Biomed. Opt. Express 10, 5952–5973 (2019). BOEICL 2156-7085 10.1364/BOE.10.005952 31799057 PMC6865125

[r13] White B. et al., “Wavelength censoring for spectroscopy in optical functional neuroimaging,” Phys. Med. Biol. 66, 065026 (2021). PHMBA7 0031-9155 10.1088/1361-6560/abd418 33326946 PMC8059274

[14] Padawer-Curry J. et al., “Variability in atlas registration of optical intrinsic signal imaging and its effect on functional connectivity analysis,” J. Opt. Soc. Am. A 38, 245–252 (2021). JOAOD6 0740-3232 10.1364/JOSAA.410447 PMC7993363 33690536

[15] White B. et al., “Statistical approaches to temporal and spatial autocorrelation in resting-state functional connectivity in mice measured with optical intrinsic signal imaging,” Neurophotonics 9, 041405 (2022). 10.1117/1.NPh.9.4.041405 35295407 PMC8920489

[16] Clarke J. et al., “SIRT1 mediates hypoxic post-conditioning-and resveratrol- induced protection against functional connectivity deficits after subarachnoid hemorrhage,” J. Cereb. Blood Flow Metab. 42, 1210–1223 (2022). JCBMDN 0271-678X 10.1177/0271678X221079902 35137611 PMC9207494

[17] Zhang X. et al., “Automated sleep state classification of wide-field calcium imaging data via multiplex visibility graphs and deep learning,” J. Neurosci. Methods 366, 109421 (2022). JNMEDT 0165-0270 10.1016/j.jneumeth.2021.109421 34822945 PMC9006179

[18] Worsley K. et al., “A unified statistical approach for determining significant signals in images of cerebral activation,” Hum. Brain Mapp. 4, 58–73 (1996). 10.1002/(SICI)1097-0193(1996)4:1<58::AID-HBM4>3.0.CO;2-O 20408186

[19] Worsley K. et al., “A three-dimensional statistical analysis for CBF activation studies in human brain,” J. Cereb. Blood Flow Metab. 12, 900–918 (1992). JCBMDN 0271-678X 10.1038/jcbfm.1992.127 1400644

[20] Chen G. et al., “A tail of two sides: artificially doubled false positive rates in neuroimaging due to the sidedness choice with *t*-tests,” Hum. Brain Mapp. 40, 1037–1043 (2019). 10.1002/hbm.24399 30265768 PMC6328330

[21] Eklund A. Knutsson H. Nichols T., “Reply to Chen et al.: parametric methods for cluster inference perform worse for two-sided t-tests,” Hum. Brain Mapp. 40, 1689–1691 (2019). 10.1002/hbm.24465 30537343 PMC6491977

[22] Worsley K., “Local maxima and the expected Euler characteristic of excursion sets of $\chi^{2}$, f and t fields,” Adv. Appl. Probab. 26, 13–42 (1994). AAPBBD 0001-8678 10.2307/1427576

[23] Ashby G., Personal Communication, University of California Santa Barbara (8 August 2022).

[24] Friston K. et al., “Assessing the significance of focal activations using their spatial extent,” Hum. Brain Mapp. 1, 210–220 (1994). 10.1002/hbm.460010306 24578041

[25] Cao J., “The size of the connected components of the excursion sets of $\chi^{2}$, t and f fields,” Adv. Appl. Probab. 31, 579–595 (1999). AAPBBD 0001-8678 10.1239/aap/1029955192

[26] Hayasaka S. Nichols T., “Validating cluster size inference: random field and permutation methods,” NeuroImage 20, 2343–2356 (2003). NEIMEF 1053-8119 10.1016/j.neuroimage.2003.08.003 14683734

[27] Brier L., Personal Communication, Washington University in St. Louis (25 July 2022).

[28] Worsley K. et al., “A general statistical analysis for fMRI data,” NeuroImage 15, 1–15 (2002). NEIMEF 1053-8119 10.1006/nimg.2001.0933 11771969

[29] Xiong J. et al., “Clustered pixels analysis for functional MRI activation studies of the human brain,” Hum. Brain Mapp. 3, 287–301 (1995). 10.1002/hbm.460030404

[30] Keibel S. et al., “Robust smoothness estimation in statistical parametric maps using standardized residuals from the general linear model,” NeuroImage 10, 756–766 (1999). NEIMEF 1053-8119 10.1006/nimg.1999.0508 10600421

[31] Holmes A. et al., “Nonparametric analysis of statistic images from functional mapping experiments,” J. Cereb. Blood Flow Metab. 16, 7–22 (1996). JCBMDN 0271-678X 10.1097/00004647-199601000-00002 8530558

[32] Nichols T. Holmes A., “Nonparametric permutation tests for functional neuroimaging: a primer with examples,” Hum. Brain Mapp. 15, 1–25 (2001). 10.1002/hbm.1058 PMC6871862 11747097

[33] Arridge S. Cope M. Delpy D., “The theoretical basis for the determination of optical pathlengths in tissue: temporal and frequency analysis,” Phys. Med. Biol. 37, 1531–1560 (1992). PHMBA7 0031-9155 10.1088/0031-9155/37/7/005 1631197

[34] Waters J., “Sources of widefield fluorescence from the brain,” eLife 9, e59841 (2020). 10.7554/eLife.59841 33155981 PMC7647397

[35] Schwartzmann A. Telschow F., “Peak p-values and false discovery rate inference in neuroimaging,” NeuroImage 197, 402–413 (2019). NEIMEF 1053-8119 10.1016/j.neuroimage.2019.04.041 31028923 PMC6752954

