# Supplementary material for: Controlling the familywise error rate in widefield optical neuroimaging of functional connectivity in mice
Source: Neurophotonics. 2023 Feb 3;10(1):015004. doi: 10.1117/1.NPh.10.1.015004 (PMC9896098; doi:10.1117/1.NPh.10.1.015004)
Supplement: Supplementary file 1 [file NPh_010_015004_SD001.pdf]

Supplemental Methods

At the request of reviewers, we here include a listing of differences between our algorithm and the software toolbox of Brier & Culver [7]. At the time of writing of the manuscript, the most recent code version was dated 4/5/21. We contacted Lindsay Brier on 7/31/22 with some of the major bugs described in the manuscript [26], which were fixed with an updated code release on 8/9/22.

We have only examined the programs in the package relevant to group differences in functional connectivity maps. The main program of relevance is `Mouse_Master_FC.m` which calls `cluster_threshold.m` (on line 100). In turn, `cluster_threshold.m` first calls `FWHM_SpAut.m` (line 7), which calculates a FWHM using the spatial autocorrelation method. On line 45, the FWHM calculation includes the origin twice. The correct formula is `FWHM=2*(row_f(1)-1)-1`. This error appears to have been fixed in the recent update.

Returning to `cluster_threshold.m`, line 11 calculates the number of resels in the entire field-of-view rather than the segmented brain. Line 14 calculates the Euler characteristic for a Gaussian random field in two dimensions. This equation should use the equation for a *t*-distribution random field, and it should include the contributions from zero and one dimensional resels. This equation also assumes one-sided inference (the Euler characteristic should be twice as large). On line 23, they calculate Beta (which is the equivalent of our λ) using the simplified equation, which is only valid for large T and Gaussian fields. In the 4/5/21 release there is an extra power of $\Gamma(2)$ in this equation, which has been removed on 8/9/21 (note $\Gamma(2)=1$, so this had no practical effect).

After calculating the cluster size threshold, `Mouse_Master_FC.m` calls `FC_ttest.m`, which performs a *t*-test using the MATLAB function `ttest.m` (line 69) or `ttest2.m` (line 56), which by default are two-tailed and calculate significance with an alpha level of 0.05. Note that this sidedness does not match how the RFT threshold was calculated. The MATLAB map of significant pixels (at p=0.05) is then used to form clusters rather than the T threshold used to calculate the cluster threshold. This code was changed in the 8/9/22 release to have `ttest.m` and `ttest2.m` threshold at a requested alpha level, which should correspond to the T threshold. We have not tested whether this new code works as intended; it is simpler to directly threshold the *t*-statistic image output by `ttest.m` or `ttest2.m` rather than use the MATLAB significance matrix, as is done here.
